# Progression and Longitudinal Biometric Changes in Highly Myopic Eyes

**DOI:** 10.1167/iovs.61.4.34

**Published:** 2020-04-25

**Authors:** Jonathan Tak Loong Lee, Xinxing Guo, Zhixi Li, Monica Jong, Padmaja Sankaridurg, Mingguang He

**Affiliations:** 1 State Key Laboratory of Ophthalmology, Zhongshan Ophthalmic Center, Sun Yat-sen University, Guangzhou, China; 2 Centre for Eye Research Australia; Ophthalmology, Department of Surgery, University of Melbourne, Melbourne, Australia; 3 Wilmer Eye Institute, Johns Hopkins University, Baltimore, Maryland, United States; 4 Brien Holden Vision Institute, Sydney, Australia; 5 School of Optometry and Vision Science, University of New South Wales, New South Wales, Australia

**Keywords:** high myopia, progression, spherical equivalent refraction, axial length, longitudinal study

## Abstract

**Purpose:**

To examine 2-year progression rate and associated biometric changes in highly myopic eyes.

**Methods:**

This is a longitudinal, observational cohort study that included 657 participants aged 7 to 70 years with bilateral high myopia (≤−6.00 diopters [D]) and followed for 2 years. All participants underwent ocular biometry and cycloplegic refraction examinations. Main outcome measures were changes in spherical equivalent refraction (SE) and ocular biometry in the right eyes.

**Results:**

Mean age of participants was 21.6 ± 12.2 years. At baseline, mean SE was −9.82 ± 3.28 D and ocular biometric measurements were 27.40 ± 1.56 mm for axial length, 3.16 ± 0.27 mm for anterior chamber depth, 3.60 ± 0.35 mm for lens thickness, and 20.09 ± 1.50 mm for vitreous chamber depth. After 2 years of follow-up, there was a trend toward more myopia and greater axial elongation in all age groups. Younger participants (≤20 years) had significantly (*P* < 0.001) greater rates of myopic shift and axial elongation compared with older participants (>20 years). However, highly myopic adults aged 40 to 70 years continued to demonstrate refractive progression, particularly if they had extremely high myopia (≤−10.00 D). In the multiple regression analysis, each additional diopter of myopia at baseline was associated with a 11% higher risk of a >1.00-D/y myopic shift (odds ratio, 1.11; 95% confidence interval, 1.04–1.18; *P* = 0.002).

**Conclusions:**

Longitudinal data from this large Chinese cohort suggest that highly myopic eyes continue to progress in SE throughout life, with the greatest rates of progression observed in younger participants. Axial elongation rates appeared to stabilize after 20 years of age and were predominantly due to an increase in the vitreous chamber depth. Other risk factors for a myopic shift included a higher degree of myopic refraction at baseline.

The boom of myopia has affected populations worldwide,^1^^–^^3^ with an accompanying rise in the prevalence of high myopia, especially among Asian countries.^4^^–^^6^ This myopia epidemic is particularly problematic in the developed regions of East and Southeast Asia, where high myopia is estimated to affect up to 21% of urban university-aged students in China,^7^ Taiwan,^4^ Korea,^8^ and Singapore.^9^

The visual impairment from high myopia and its associated complications, which include retinal detachment, glaucoma, cataract, and myopic maculopathy,^10^^,^^11^ are a major cause of legal blindness in many developed countries.^12^^–^^14^ Especially for younger individuals, the loss of productive working years, lower quality of life, and cost of correcting refractive error have large financial implications and impose a heavy socioeconomic burden worldwide.^15^^–^^17^

The severity of refractive error, presence of ocular deformity, and increasing age appear to be important risk factors for the development of pathologic changes in myopia.^18^^–^^20^ According to previous studies of highly myopic eyes, deepening of the vitreous chamber correlates with worsening spherical equivalent refraction (SE),^21^^–^^23^ suggesting that vision-threatening complications such as chorioretinal atrophy and choroidal neovascularization may be related to biomechanical stretch that is focused on the posterior segment of the globe.^24^^–^^26^

Although it is clear that pathologic myopia is not a static disease,^27^ the progression of ocular biometry in highly myopic eyes has not been well described in the literature. Results from longitudinal studies of high myopes provide evidence that axial length (AL) continues to elongate even in the fourth decade of life, but many of these studies were limited by a lack of young school-aged participants,^28^^–^^30^ while those that included teenagers were constrained by incomplete ocular biometry measurements.^31^ In the present study, the aim was to examine the progression of refractive error and ocular components over a 2-year period in a large cohort of highly myopic Chinese children and adults. Using data from the Zhongshan Ophthalmic Center–Brien Holden Vision Institute (ZOC-BHVI) High Myopia Cohort registry, we analyzed a range of biometric parameters to explore the relationship of high myopia progression with baseline refraction, biometry, and age.

## Methods

### Study Participants

Participants were recruited from the ZOC-BHVI High Myopia Cohort Study, which has been described in detail elsewhere.^32^ In brief, patients aged 7 to 70 years with bilateral myopic sphere measuring −6.00 diopter (D) or greater were recruited from the Department of Optometry and community screening program at ZOC in Guangzhou. Participants were excluded if they had a history of autoimmune disease, secondary myopia, previous refractive or intraocular surgery, significant optic media opacity preventing fundus examination (e.g., dense corneal opacity), or severe systemic health conditions that precluded follow-up. A baseline examination was conducted on 884 patients from November 2011 to October 2012 in the Clinical Research Center at ZOC. Of these, 657 patients (74.3%) participated in the 2-year follow-up visit (2013–2015), while the remaining 227 persons declined or were lost to follow-up.

Informed written consent was obtained from all participants, including from parents or guardians of those under 18 years. This study adhered to the tenets of the Declaration of Helsinki, and ethical approval was obtained from the Institutional Review Board of ZOC.

### Ophthalmic Examinations

Ocular biometry including AL, anterior chamber depth (ACD), and lens thickness (LT) were measured by the Lenstar LS900 (Haag-Streit AG, Koeniz, Switzerland) before cycloplegia in a room with low ambient light. If the AL exceeded the Lenstar measurement range of 32 mm, then the IOL Master (Carl Zeiss Meditec, Oberkochen, Germany) was used. Three consecutive biometry readings were taken for each eye. Refraction was measured using an autorefractor (Topcon KR8800; Topcon Corp, Tokyo, Japan) after cycloplegia. Cycloplegia was induced with two drops of 0.5% tropicamide and 0.5% phenylephrine (Mydrin-P; Santen Pharmaceutical Co. Ltd., Osaka, Japan), administered 5 minutes apart to each eye. After an additional 10 to 20 minutes, cycloplegia was considered complete if the pupil was ≥6 mm in diameter and unreactive to light.

### Statistical Analysis

Statistical analysis was performed using Stata software V12.0 (Stata, College Station, TX). Spherical equivalent refraction, defined as the sum of the spherical power +1/2 cylindrical power, was used to calculate the refractive error in diopters. Negative direction SE changes were considered a myopic shift. Age was defined as the age at baseline examination. For the distribution of biometric progression, participants were further categorized into six groups: 7 to 10 years, 11 to 20 years, 21 to 30 years, 31 to 40 years, 41 to 50 years, and 51 to 70 years. Each age group was further subdivided into three ranges by SE: −8.00 to ≤−6.00 D, −10.00 to ≤−8.00 D, and ≤−10.00D. Vitreous chamber depth (VCD) was calculated by subtracting the central corneal thickness (CCT), ACD, and LT from the AL (VCD = AL – CCT – ACD – LT). Right eyes were arbitrarily chosen for analysis. Comparisons of categorical variables across groups were performed using Pearson's chi-squared test. Comparisons of continuous variables across groups were performed using two-sample *t* tests or ANOVA tests, depending on the number of groups of interest.

Multiple logistic regression was used to analyze the association of myopic shift and axial elongation with age, sex, baseline SE, and baseline AL. Odds ratios (ORs) with 95% confidence intervals (CIs) were calculated.

## Results

Of the 884 patients who completed the baseline examination, a total of 657 participants (74.3%) had at least one recorded refractive or biometric value at the 2-year follow-up, enabling them to participate in the longitudinal study. An overview of the demographic and biometric characteristics of the right eyes of participants and nonparticipants at the baseline examination is summarized in Table 1. Participants of the follow-up visit (*n* = 657) were significantly younger (*P* < 0.001) and less myopic (*P* < 0.001) than nonparticipants (*n* = 227), with a corresponding shorter mean AL (*P* < 0.001) and shallower mean VCD (*P* = 0.015). Both groups did not vary significantly with regard to sex (*P* = 0.61) or ACD (*P* = 0.48). A locally weighted scatterplot smoothing model of AL and SE change was plotted against age (Supplementary Figures S1 and S2, respectively), which revealed that the trend of biometric change clearly shifted at approximately 20 years of age. There were 407 participants aged ≤20 years (61.9%), and the remaining 250 participants were aged >20 years (38.1%).

**Table 1.** Baseline Characteristics of Participants and Nonparticipants of the 2-Year Follow-up Examination

Characteristic	Participants (*n* = 657)	Nonparticipants (*n* = 227)	*P* Value
Females, %	53.1	55.1	0.61^*^
Age (years)	21.6 ± 12.2	26.1 ± 12.6	<0.001^†^
7–10	39 (5.9)	8 (3.5)	
11–20	368 (56.0)	76 (33.5)	
21–30	131 (19.9)	77 (33.9)	
31–40	55 (8.4)	34 (15.0)	
41–50	36 (5.5)	19 (8.4)	
50–70	28 (4.3)	13 (5.7)	
SE (D)	−9.82 ± 3.28	−11.00 ± 4.47	<0.001^†^
AL (mm)	27.40 ± 1.56	27.87 ± 1.77	<0.001^†^
CCT (µm)	539.39 ± 36.04	545.51 ± 33.23	<0.001^†^
ACD (mm)	3.16 ± 0.27	3.14 ± 0.29	0.48^†^
LT (mm)	3.60 ± 0.35	3.69 ± 0.37	0.001^†^
VCD (mm)	20.09 ± 1.50	20.39 ± 1.68	0.015^†^

Values are presented as number (%) or mean ± SD unless otherwise indicated.

* Comparison underwent Pearson's chi-squared test.

† Comparison underwent two-sample *t* test.

### Myopia and Ocular Biometry Progression by Age, Sex, SE, and AL

The annual change in SE and ocular biometry according to age, sex, SE, and AL at baseline is shown in Table 2. The number of participants with available refractive or biometric data for each parameter is listed. There was a mean myopic shift of −0.47 D/y (95% CI, −0.51 to −0.43, *n* = 643) across all age groups. Younger participants aged ≤20 years were significantly (two-sample *t* test, *P* < 0.001) more likely to become more myopic (−0.56 D/y; 95% CI, −0.60 to −0.51; *n* = 405) than older participants aged >20 years (−0.32 D/y; 95% CI, −0.39 to −0.25; *n* = 238). The largest myopic shifts were observed in those aged 7 to 10 years (−0.65 D/y; 95% CI, −0.82 to −0.48; *n* = 39), with a gradual decline to the smallest myopic shift, which occurred in the 31- to 40-year age group (−0.24 D/y; 95% CI, −0.40 to −0.09; *n* = 52). Beyond 40 years, the rate of myopic shift increased again, with the oldest group aged 51 to 70 years recording −0.53 D/y (95% CI, −0.78 to −0.29; *n* = 27). A statistically significant (two-sample *t* test, *P* < 0.001) pattern was observed for annual AL change across the age groups, with greatest elongation observed in the younger participants ≤20 years (0.19 mm/y; 95% CI, 0.18 to 0.21; *n* = 380), as compared with those aged >20 years (0.05 mm/y; 95% CI, 0.04 to 0.07; *n* = 225). Overall, there was a mean increase in the LT (0.04 mm/y; 95% CI, 0.04 to 0.04, *n* = 584), with a corresponding reduction in the ACD (−0.03 mm/y; 95% CI, −0.04 to −0.03, *n* = 590). Axial elongation appeared to be largely attributable to an increase in the VCD (all-age means for annual change in AL and VCD were both 0.14 mm/y; 95% CI, 0.13 to 0.15), rather than changes in the ACD or LT. The results do not show any significant differences between males and females with regard to annual change in SE or ocular biometry (*P* > 0.05 for all).

**Table 2.** Annual Stratified Rate Change for Spherical Equivalent Refraction and Ocular Biometry as a Function of Baseline Characteristics

	Annual Change				
	SE (D)	AL (mm)	ACD (mm)	LT (mm)	VCD (mm)
	Mean (95% CI)	Mean (95% CI)	Mean (95% CI)	Mean (95% CI)	Mean (95% CI)
Characteristic	*n* = 643	*n* = 605	*n* = 590	*n* = 584	*n* = 580
Baseline age (years)					
7–10	−0.65 (−0.82 to −0.48)	0.31 (0.25 to 0.37)	−0.01 (−0.02 to 0.00)	0.01 (0.00 to 0.02)	0.31 (0.26 to 0.37)
11–20	−0.55 (−0.60 to −0.50)	0.18 (0.16 to 0.20)	−0.04 (−0.04 to −0.03)	0.04 (0.03 to 0.04)	0.18 (0.16 to 0.19)
21–30	−0.28 (−0.37 to −0.19)	0.05 (0.03 to 0.06)	−0.05 (−0.06 to −0.04)	0.05 (0.04 to 0.06)	0.04 (0.03 to 0.06)
31–40	−0.24 (−0.40 to −0.09)	0.06 (0.04 to 0.09)	−0.03 (−0.04 to −0.02)	0.04 (0.03 to 0.04)	0.05 (0.03 to 0.08)
41–50	−0.40 (−0.62 to −0.17)	0.07 (0.05 to 0.10)	−0.03 (−0.04 to −0.02)	0.04 (0.03 to 0.04)	0.07 (0.05 to 0.10)
51–70	−0.53 (−0.78 to −0.29)	0.06 (0.03 to 0.08)	0.02 (−0.04 to 0.09)	0.02 (0.01 to 0.03)	0.05 (0.02 to 0.09)
*P* value	<0.001^*^	<0.001^*^	<0.001^*^	<0.001^*^	<0.001^*^
All	−0.47 (−0.51 to −0.43)	0.14 (0.13 to 0.15)	−0.03 (−0.04 to −0.03)	0.04 (0.04 to 0.04)	0.14 (0.13 to 0.15)
Sex					
Male	−0.50 (−0.56 to −0.44)	0.14 (0.13 to 0.16)	−0.03 (−0.04 to −0.03)	0.04 (0.04 to 0.04)	0.14 (0.13 to 0.16)
Female	−0.44 (−0.49 to −0.39)	0.14 (0.12 to 0.16)	−0.03 (−0.04 to −0.03)	0.04 (0.03 to 0.04)	0.14 (0.12 to 0.15)
*P* value	0.15^†^	0.75^†^	0.68^†^	0.19^†^	0.59^†^

The right eyes of patients were analyzed in the study.

† Comparison underwent two-sample *t* test.

* Comparison underwent ANOVA test.

**Figure 1.**
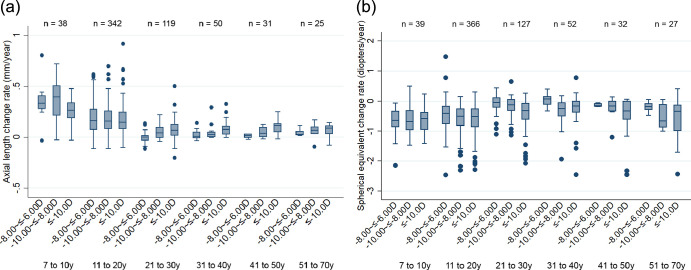
Boxplots showing the annual rate of (a) axial length and (b) spherical equivalent refraction change according to baseline age and spherical equivalent refraction.

Figure 1 shows the annual rate of (a) AL and (b) SE change, stratified according to age group and severity of myopia at baseline. The greatest range and rates of AL change were observed in the younger age groups (7 to 10 years and 11 to 20 years) (Fig. 1a). In the adults over 20 years of age, there was less axial elongation per year, although eyes measuring ≤−10.00 D had a higher median AL change rate (median for 21–30 years, 0.07 mm/y; 31–40 years, 0.08 mm/y; 41–50 years, 0.11 mm/y; 51–70 years, 0.09 mm/y), compared with those with less severe high myopia of −8.00 to ≤−6.00 D (median for 21–30 years, 0 mm/y; 31–40 years, 0.01 mm/y; 41–50 years, 0.02 mm/y; 51–70 years, 0.03 mm/y). The data on refractive change demonstrate that in the least severe high myopia subgroup (−8.00 to ≤−6.00 D), the younger participants had a higher median rate of SE change toward myopia (median for 7–10 years, −0.65 D/y; 11–20 years, −0.41 D/y) compared with the adults over the age of 20 (median for 21–30 years, −0.05 D/y; 31–40 years, +0.07 D/y; 41–50 years, −0.16 D/y; 51–70 years, −0.18 D/y) (Fig. 1b). Adults with a baseline refraction of −10.00 D or worse continued to demonstrate an appreciable myopic shift (median for 21–30 years, −0.32 D/y; 31–40 years, −0.16 D/y; 41–50 years, −0.33 D/y; 51–70 years, −0.34 D/y).

**Figure 2.**
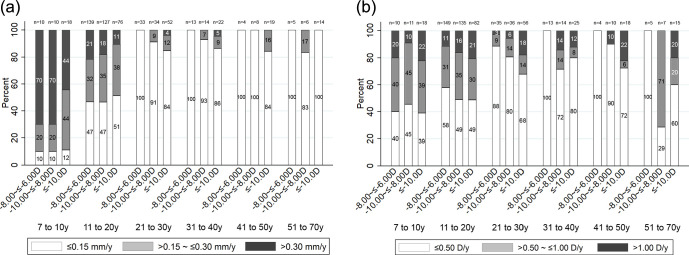
Distribution of (a) axial elongation and (b) myopic shift per year according to baseline age and spherical equivalent refraction.

Figure 2 divides participants based on the amount of (a) axial elongation and (b) myopic shift per year, stratified according to age group and severity of myopia at baseline. There was an age-dependent decrease in the proportion of eyes with greater than 0.30 mm axial elongation per year, from 57.9% (*n* = 22/38) in the 7- to 10-year age group, to 17.8% (*n* = 61/342) in the 11- to 20-year age group, 1.8% (*n* = 3/168) in those aged 21 to 40 years, and none in those aged greater than 40 years (Fig. 2a). Myopia progression of greater than 1.00 D per year was observed in approximately 15% (*n* = 63/405) of the participants younger than 20 years (Fig. 2b). An equivalent myopic shift occurred in 10.2% (*n* = 13/127) of the 21- to 30-year age group and 9.6% (*n* = 5/52) of the 31- to 40-year age group. After the age of 40 years, the proportion of participants with a >1.00-D per year myopic progression increased again to 13.6% (*n* = 8/59). Among the patients aged 40 years and older stratified by baseline refraction, a myopic shift of greater than 1.00 D per year was more common in participants measuring ≤−10.00 D (21.2%, *n* = 7/33) at baseline compared with those measuring −10.00 to ≤−8.00 D (5.9%, *n* = 1/17) or −8.00 to ≤−6.00 D (none) at baseline (Fig. 2b).

### Associated Risk Factors for SE and AL Progression

To perform multiple logistic regression, a cutoff value of >1.00 D/y for SE progression was selected because it represents a clinically significant myopic shift whereby a new prescription is likely to be required for the patient. Accordingly, significant axial elongation was defined as a cutoff value of >0.30 mm/y, after direct conversion of the myopic shift cutoff value (1 D divided by the ratio between AL change and SE change). In the multiple regression analysis for myopic shift, SE progression of >1.00 D/y was significantly associated with younger age (OR, 0.97; 95% CI, 0.95–0.995; *P* = 0.018) and more severe baseline myopic refraction (OR, 1.11; 95% CI, 1.04–1.18; *P* = 0.002) (Table 3). Sex did not have a significant effect on refractive progression (*P* > 0.05).

**Table 3.** Logistic Regression to Determine the Association Between Myopic Shift, Axial Length, and Baseline Characteristics

	Myopic Shift >1.00 D/y^*^			Axial Length Elongation >0.30 mm/y^†^		
Characteristic	OR	95% CI	*P* Value	OR	95% CI	*P* Value
Baseline age (years)	0.97	0.95–0.995	0.018	0.68	0.62–0.74	<0.001
Sex	0.77	0.49–1.21	0.258	1.48	0.85–2.59	0.162
Baseline SE (D)	1.11	1.04–1.18	0.002			
Baseline AL (mm)				1.34	1.08–1.67	0.007

* Multiple logistic regression and included age, sex, and baseline SE as independent variables.

† Multiple logistic regression and included age, sex, and baseline AL as independent variables.

In the multiple regression analysis for axial elongation, AL progression of >0.30 mm/y was significantly associated with younger age (OR, 0.68; 95% CI, 0.62–0.74; *P* < 0.001) and greater baseline axial length (OR, 1.34; 95% CI, 1.08–1.67; *P* = 0.007) (Table 3).

## Discussion

In this study, the longitudinal progression of refraction and ocular biometry was investigated for a clinic-based cohort of 657 highly myopic Chinese patients over a 2-year period. There was a trend toward a myopic shift and greater axial elongation across all participants aged between 7 and 70 years. Greater myopic shifts were observed in younger participants and those with more myopic baseline refraction. Axial elongation was most pronounced in participants under the age of 20 years (0.19 mm/y). Importantly, adults with high myopia also continued to show signs of AL and SE progression.

High myopia progression was greatest in participants aged ≤20 years at baseline (−0.56 D/y), particularly for children aged 7 to 10 years (−0.65 D/y). Donovan and colleagues^33^ reported comparable results in a meta-analysis of 20 studies of low-to-moderate myopia in urban children of similar age (7–12 years), estimating myopia progression to be between −0.55 D and −0.82 D/y, with the rate of progression decelerating with age. Due to the rarity of high myopia in this early age group,^34^ there have been few studies documenting incidence and progression before the age of 11 years. The Singapore Cohort of the Risk Factors for Myopia analysis of 273 myopic children aged 7 to 9 years found an increased risk of high myopia with younger age of myopia onset, with the overall prevalence of high myopia increasing fivefold (from 3.3% to 16.8%) over a period of 3 years.^35^ In a Danish clinic-based study of 14-year-old adolescents (*n* = 36) with ≤−6.00-D high myopia followed for 40 years, Goldschmidt et al.^31^ found that mean progression of high myopia was greatest in the first 10 years (mean change, 2.09 D), but direct comparisons are difficult as the study lacked younger children and did not examine a high prevalence region such as East Asia. In general, a younger age of high myopia onset is associated with a longer duration of disease, and therefore children who are already beyond −6.00 D before the age of 11 years are at great risk of developing vision-threatening myopic complications during their productive years.^5^^,^^36^

When examining the young adults (20 to 40 years) in our study, we found that myopic progression dropped to a rate of −0.24 to −0.28 D/y. Interestingly, in the 40- to 70-year age group, there was a resurgent increase in myopic shift (−0.40 D/y in those 41–50 years and −0.53 D/y in those 51–70 years), and although there is continued axial elongation (0.06 to 0.07 mm/y), this is not sufficient to explain the degree of refractive progression, suggesting that other factors such as early nuclear cataract may be contributing to myopic progression in this older high myopia population.^37^^,^^38^ Furthermore, progression in adults was more pronounced in those with severe high myopia (≤−10.00 D) at baseline, suggesting that there may be two distinct populations of high myopia in this cohort. Many of the adult eyes from the least myopic distribution of the sample (−8.00 to ≤−6.00 D) appeared to be refractively stable. These eyes may represent the worst cases of the physiologic spectrum of school myopia and would be expected to stabilize by the third decade of life and maintain a good visual prognosis.^39^ In contrast, a subset of adult participants with severe high myopia (≤−10.00 D) at baseline continued to progress throughout adulthood. In support of these findings, Saka et al.^28^ examined 185 highly myopic adult eyes (mean age 48.4 ± 12.2 years) and reported that the mean refractive error became more myopic (−13.0 ± 4.3 D to −13.5 ± 4.3 D) after 2 years of follow-up. This adult group with more severe high myopia may represent a continuum of more aggressive, potentially genetically influenced pathologic myopia that begins in early childhood and eventually results in the time-dependent complications such as myopic maculopathy and posterior staphyloma.^5^^,^^18^ There is support for this idea from a recent retrospective case series of 29 children at a Japanese high myopia clinic, whereby participants under the age of 15 years with a mean refractive error of –9.6 ± 4.8 D were followed for over 20 years.^40^ At the last visit, over half (63%) of eyes met the definition of pathologic myopia in adulthood, of which most (83%) had evidence of diffuse chorioretinal atrophy in childhood. This suggests that adult patients who go on to develop pathologic myopia may already exhibit unique fundus changes in early childhood that are obviously different from the fundi of children with school myopia.

The AL of the adult eye in population-based cross-sectional studies has previously been reported to decrease with increasing age,^37^^,^^38^ but our results show that the eye continues to elongate at a rate of 0.05 to 0.07 mm per year in highly myopic patients aged >20 years, largely due to an increase in the VCD. Our findings are comparable to two previous studies examining AL progression using the IOL Master in highly myopic (≤–6.00 D) Japanese adults.^28^^,^^41^ Ohsugi et al.^41^ reported a mean AL increase of 0.04 mm per year in a retrospective series of 165 highly myopic eyes without macular complications (age range, 34–82 years), while Saka et al.^28^ observed a significant increase in the AL of 0.13 mm over a 2-year period in a similar adult population (age range, 22–84 years). Although the precise mechanisms linking high myopia and myopic maculopathy are not clear, there is a higher prevalence of pathologic signs at greater axial lengths.^18^^,^^22^ The increased depth of the vitreous chamber with enlargement of the globe suggests that the mechanical stretch in high myopia is focused on the posterior segment, incorporating the choroid, retina, and sclera.^5^ Beyond −10.00 D, the structural integrity of the globe may be affected, and histologic and optical coherence tomography studies of highly myopic eyes confirm that there is profound thinning of the sclera and choroid in extreme myopia owing to axial elongation of the globe.^42^^,^^43^ The thinning appears to predispose eyes to macular Bruch's membrane defects, which are associated with complete loss of retinal pigment epithelium and a markedly diminished choroidal circulation with photoreceptor cell death.^44^^,^^45^ These posterior segment changes ultimately lead to the critical lesions seen in pathologic myopia, such as diffuse chorioretinal atrophy and lacquer cracks,^11^ while the reduced choroidal perfusion may lead to localized ischemia and subsequent myopic choroidal neovascularization.^46^

The major strength of this study is the large sample size of bilateral high myopia patients who prospectively underwent standardized ocular examinations. By including both children and adults, this allowed for comparisons to be drawn across a wide range of ages. Several limitations must be mentioned. Of the 884 patients who participated in the baseline examination, a total of 227 (25.7%) were lost to follow-up. Participants who attended the follow-up visit were younger (21.6 ± 12.2 years vs. 26.1 ± 12.6 years, *P* < 0.001), were less myopic (−9.82 ± 3.28 D vs. −11.00 ± 4.47 D, *P* < 0.001), and had a shorter axial length (27.40 ± 1.56 mm vs. 27.87 ± 1.77 mm, *P* < 0.001) than those who did not attend. Although attrition is an intrinsic problem in any large longitudinal epidemiologic study, this may have introduced a selection bias, and the systematic differences between participants and nonparticipants may have underestimated the overall progression rates. More than half of the participants (56.0%) belonged to a single age group (11 to 20 years), and in future studies, a larger proportion of older participants would be valuable. As the study was performed at a community-based outpatient clinic of a single institution, the results may not be generalizable to the broader population. Furthermore, patients were exclusively Chinese. Finally, the follow-up period was relatively short, and therefore the overall degree of progression was clinically insignificant for most participants. Data collection for the 4-year follow-up study is currently under way.

In conclusion, our study showed that highly myopic patients continue to progress in myopic refraction throughout life. Age appears to have the greatest influence on high myopia progression. The greatest rates of SE and AL progression were observed in younger participants aged ≤20 years, although there was a subset of severely myopic adults (≤−10.00 D) that continued to demonstrate an appreciable myopic shift, without a corresponding increase in the AL. Predictors for a myopic shift include younger age and a higher degree of myopic refraction at baseline. Axial elongation rates stabilized after 20 years of age and were predominately due to an increase in the VCD, implicating gradual biomechanical stretch of the posterior segment in the development of the pathologic lesions of high myopia. The high progression rates in the children of this study suggest that there will be a significant population of young Asian adults at risk of developing myopic maculopathy in the following decade. Strategies to slow the progression of myopia in this population to prevent the development of irreversible maculopathy are required, although regular screening of high-risk older individuals with a history of severe myopia may also be indicated.

## Supplementary Material

Supplement 1
