# Supplementary figures and images for: Progression and Longitudinal Biometric Changes in Highly Myopic Eyes
Source: Invest Ophthalmol Vis Sci. 2020 Apr 25;61(4):34. doi: 10.1167/iovs.61.4.34 (PMC7401968; doi:10.1167/iovs.61.4.34)

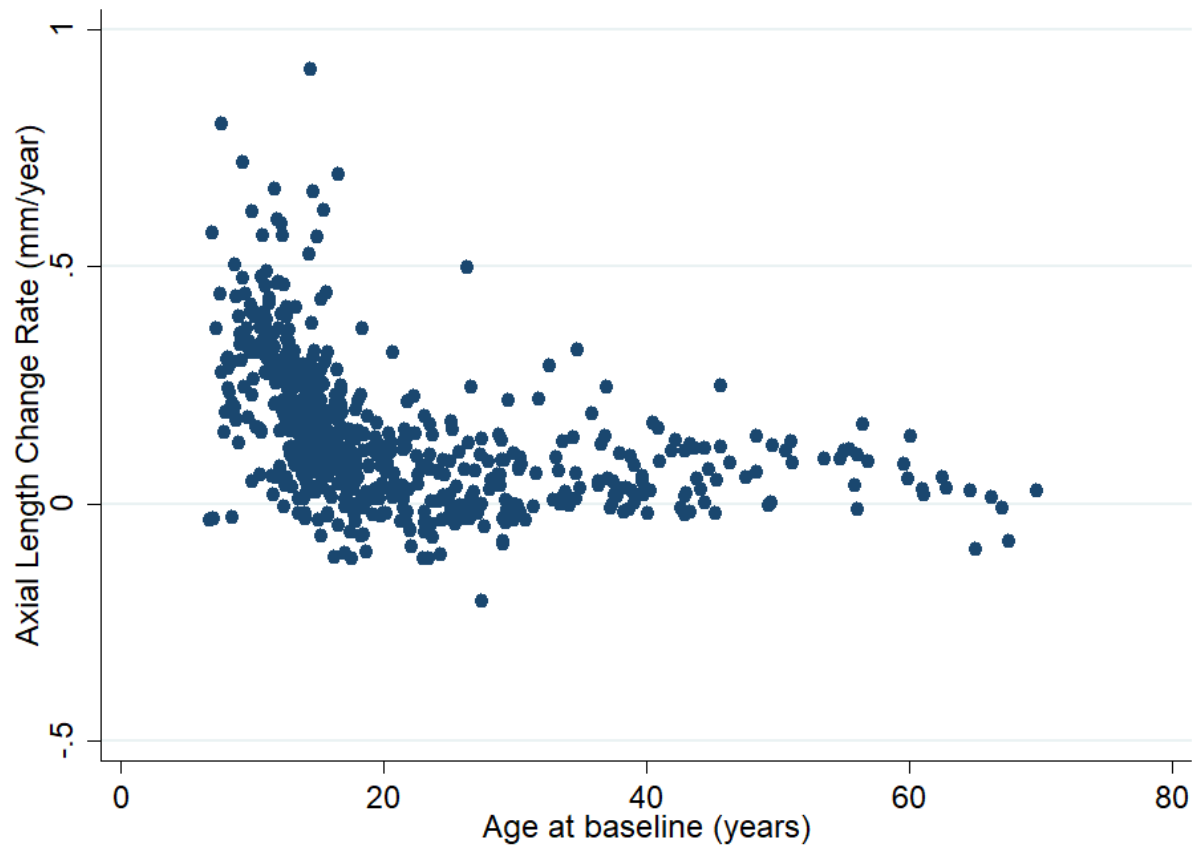

Supplementary Figure 1

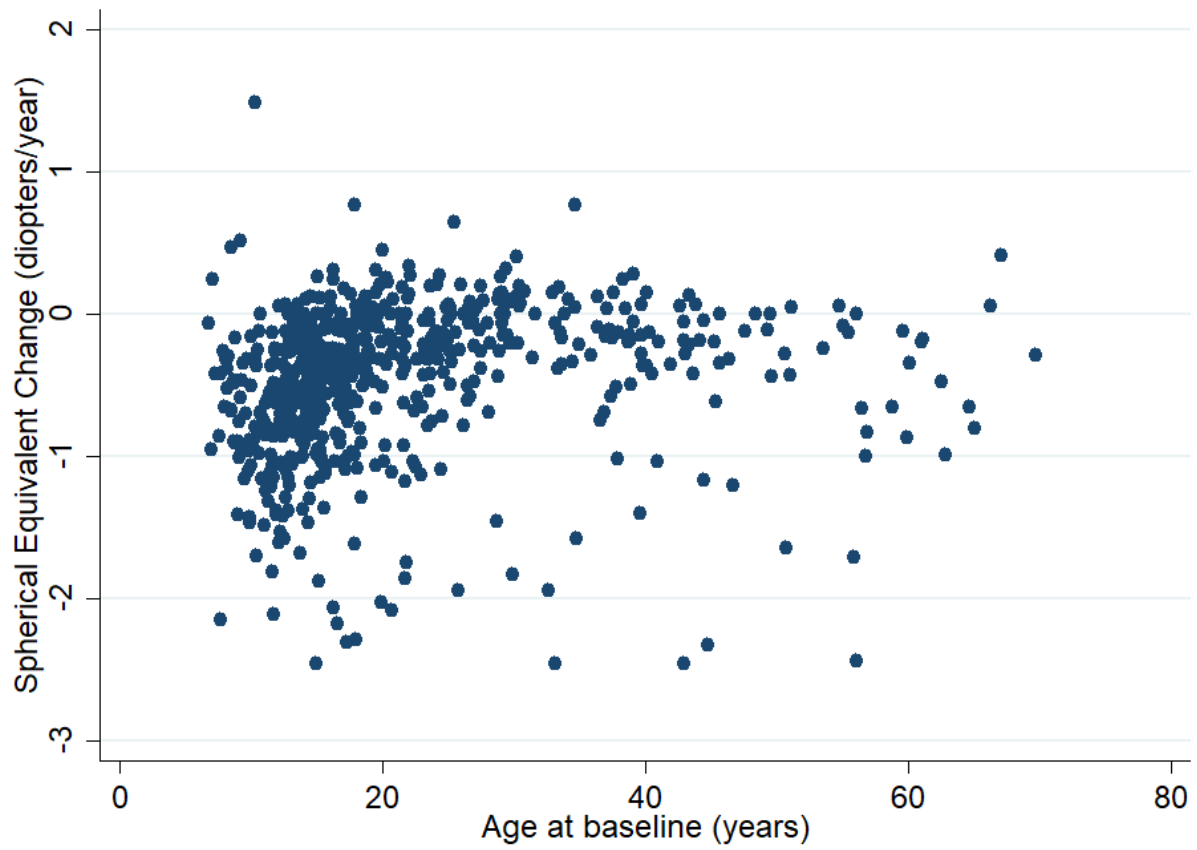

Supplementary Figure 2

Supplement: Supplement 1 [file iovs-61-4-34_s001.pdf]
